# Developmental changes in ACLs and semitendinosus tendons dimensions according to age in children

**DOI:** 10.1186/s13018-020-01845-w

**Published:** 2020-08-27

**Authors:** Ryszard Tomaszewski, Dominika Smyczek, Izabela Woś-Cieśla, Ewa Kluczewska, Tomasz Koszutski, Łukasz Wiktor

**Affiliations:** 1 Department of Pediatric Traumatology and Orthopedy, Upper Silesian Child Centre, ul. Medykow 16, 40-752 Katowice, Poland; 2 Faculty of Science and Technology, Institute of Biomedical Engineering, University of Silesia in Katowice, Katowice, Poland; 3 Department of Trauma and Orthopaedic Surgery, Upper Silesian Child Health Centre in Katowice, Silesian University of Medicine, Katowice, Poland; 4 Department of Paediatric Surgery and Urology, Upper Silesian Child Health Centre in Katowice, Silesian University of Medicine, Katowice, Poland; 5 Department of Radiology, Public Clinical Hospital in Zabrze, Silesian University of Medicine, Katowice, Poland

**Keywords:** ACL reconstruction, Hamstring tendons, Autograft, Children

## Abstract

**Purpose:**

Managing anterior cruciate ligament (ACL) injuries in skeletally immature patients remains difficult. The main aim of this study was to retrospectively compile normative data on the cross-sectional area (CSA) of the semitendinosus tendon (ST) and the diameter of the ACL in children and young adults.

**Methods:**

Knee magnetic resonance imaging (MRI) examinations were performed for a 2-year period in 132 patients (83 female and 49 male patients). The mean age was 14.9 years (8–18 years). Measurements of the ST CSA were performed on axial views in greyscale by two independent researchers. The ACL diameter was measured as well.

**Results:**

The results show the CSA of the ST was related to age, and its growth was not linear. The highest growth rate of the CSA of the ST occurred at age 12–13 at the level of the femoral growth plate and at the level of the tibial plateau. The growth of the ACL diameter was linear until 18 years of age.

**Conclusions:**

ST growth (measured in CSA increments) is almost complete at the age of 13, even though the growth is not linear. ACL growth measured in diameter increments proceeds linearly from 8 to 18 years of age. MRI is a clinically useful tool for assessing hamstring tendon grafts preoperatively.

**Level of evidence:**

Level III, diagnostic studies

## Introduction

The annual incidence of anterior cruciate ligament (ACL) injury in children and adolescents is rising steadily, and it accounts for 0.5–3% of all ACL injuries [1]. Many minors could be affected by this injury, but the available epidemiological information on ACL tears in skeletally immature patients is limited [2–5]. There are two treatment options for paediatric patients with an ACL injury, including rehabilitation only or ACL reconstruction followed by high-quality rehabilitation. This approach allows restored stability of the knee, reduced meniscal and chondral pathologies and minimized risks for femoral or tibial growth arrest in children with an ACL injury.

Clinical examination, classic radiographs and magnetic resonance imaging (MRI) are the basis for diagnosing patients and determining whether they qualify for surgical treatment [2, 6–8]. A variety of reconstructive techniques have been described, including transphyseal, physeal-sparing and partial transphyseal ACL reconstruction [6, 9]. ACL reconstruction is most commonly performed with the use of a hamstring tendon graft [2, 6]. Before surgery, preoperative planning may include an assessment of the hamstring graft diameter. For some surgeons, the evaluation of the graft dimension is based on anthropometric measurements, such as height, mass, age, sex and lower limb length [10–12]. Researchers have also suggested that computed tomography (CT), ultrasonography and MRI could be used to measure the hamstring cross-sectional area (CSA). Additionally, it has been demonstrated that this MRI measurement fully correlates with the hamstring tendon dimension determined intraoperatively [13–15]. Data collected via knee MRI were used to evaluate and characterize the growth in the semitendinosus tendon (ST) CSA and ACL diameter in children. The hypothesis was that the hamstring CSA and ACL diameter change in a non-linear manner during child development.

## Materials and methods

Knee MRI in our Department of Radiology was performed in 132 patients (83 female and 49 male patients) for a 2-year period. The mean age was 14.9 years (8–18 years). Patients were grouped according to their age, and a gap of 1 year was maintained between each group. Patients were assigned to groups according to their year of birth, and the data were analysed in this manner. In these groups, there were no patients with MRI data for both knees. The requirement for informed consent from the patient was waived. Each examination included sagittal and coronal T1- and T2-weighted and MERGE MRI sequences to obtain images adequately depicting the ACL. These sequences facilitated differentiation of the ligaments, muscles, tendons and non-ossified epiphyseal cartilage. Knee MRI examinations were separated from the tests that were performed due to medical indications. The exclusion criteria were as follows: previous arthroscopic procedure, complete or partial disruption of the ACL/posterior cruciate ligament (PCL), fractures around the knee, congenital abnormalities, knee flexion deficit greater than 15° and low-quality data with no possibility of differentiating correct anatomical structures (most commonly movement artefacts). A 1.5T HDX ECHOSPEED MRI machine was used for all of the examinations. During the MRI examination, the knee was positioned at full extension. Each study included sagittal, axial and coronal T1- and T2-weighted MRI sequences. The MRI protocols used were as follows: AX 2D MERGE for the tendon CSA and ACL diameter; -ACL T2 for the ACL diameter. Sagittal T1 FSE, sagittal Pd FRFSE +FS, coronal T2 FRFSE and other protocols were used to confirm the results. For all sequences, the section thickness was 3–5 mm, and a matrix of (256–512) × (192–256) was applied. All knees were imaged using a high-definition (HD) extremity 8-channel 2415373 S/N 951 or HD quad extremity 5147225-2S/N U 24324 coil. When necessary, the child was sedated for the examination. The following measurements were collected: age, sex, ST CSA at the level of both the femoral growth plate and tibial plateau, and ACL diameter. All numeric measurements were determined by two independent researchers (a radiologist with 8 years of experience and an orthopaedic surgeon with 18 years of experience); average measurements were analysed. The results were read using Alteris OsiriX 1.5.8. During the visualization of images and the measurement process, the researchers were blinded to the patient’s name, age and sex.

### ST measurements

**Fig. 1:**
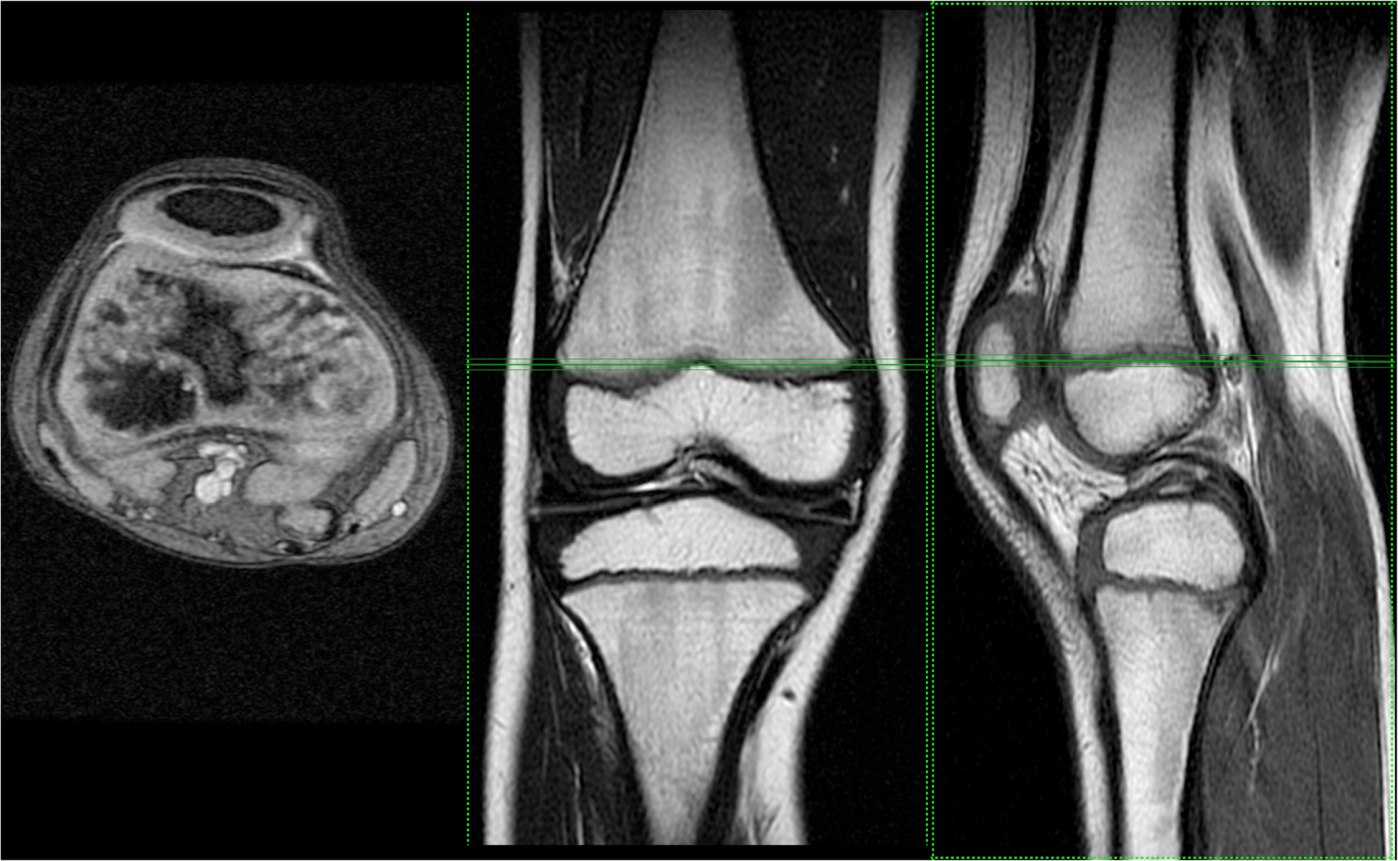
Knee MRI at the femoral growth plate level

**Fig. 2:**
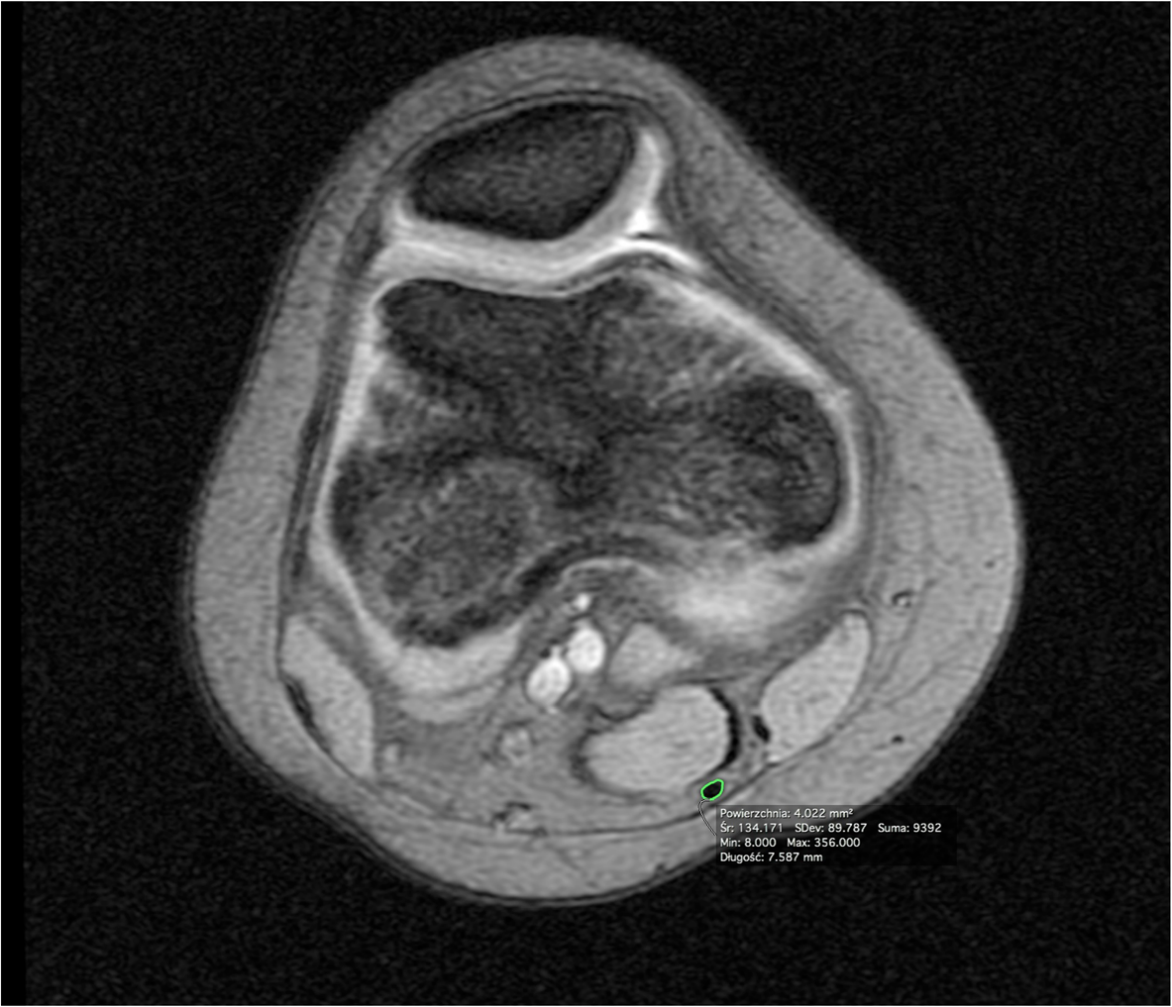
Knee MRI at the femoral growth plate level (axial view). A cross-section area of the ST tendon

The level of the femoral growth plate was determined by sagittal and frontal MRI using the SET1 sequence. At this level, in the transverse plane, the CSA of the ST was measured. The CSA of the ST was also measured on images in the transverse plane at the level of the tibial plateau. The CSA of the ST was assessed in square millimetres (Figs. 1 and 2).

### ACL measurements

**Fig. 3:**
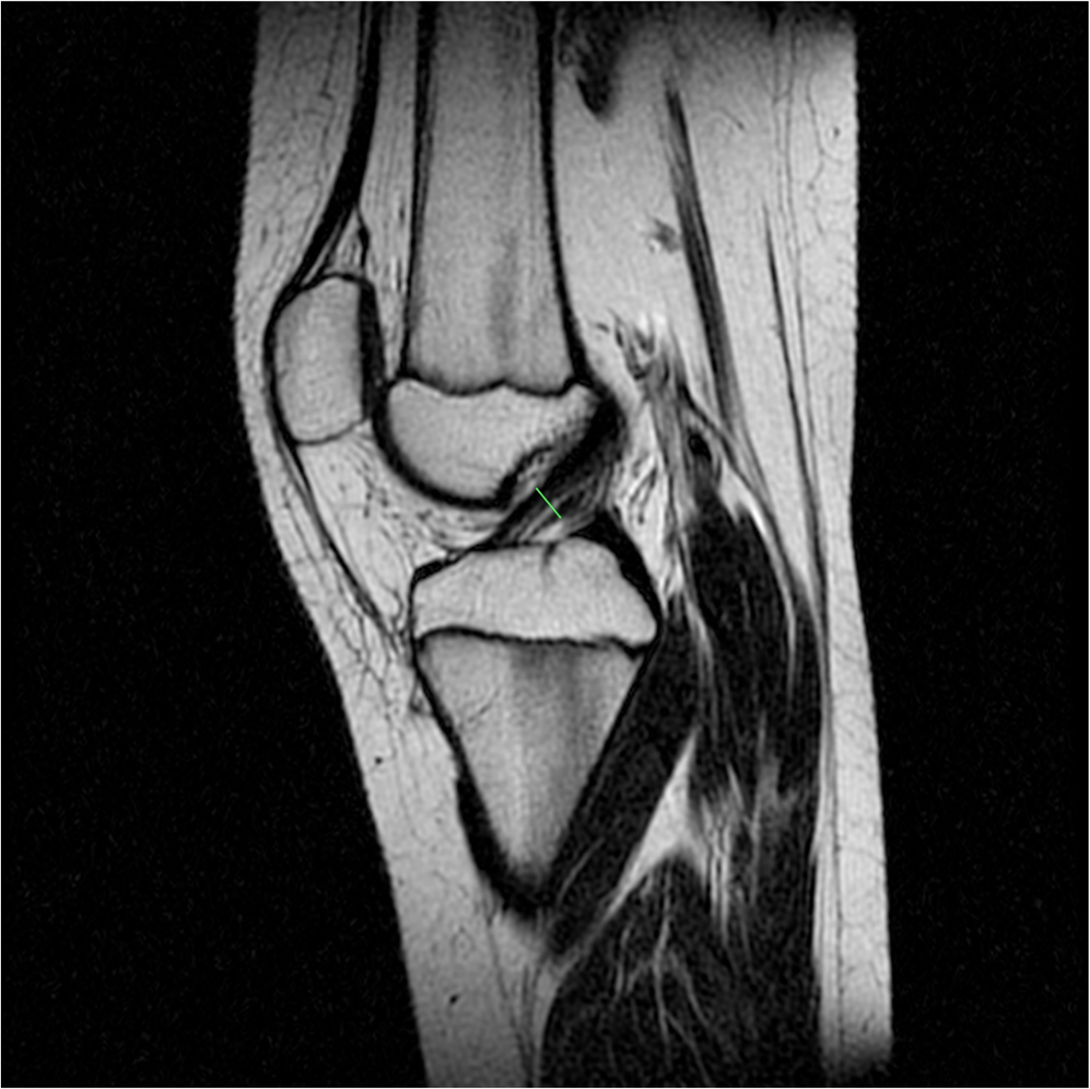
Knee MRI—measurement of the ACL diameter

The ACL diameter was measured by MRI in the sagittal plane, parallel to the course of the tendon at the thickest point in the middle of the ACL. The measurements were obtained in millimetres (Fig. 3)

### Statistical analyses

Statistical analysis was performed to determine the correlation between age (8–18 years), ST CSA (at the level of both the femoral growth plate and tibial plateau) and ACL diameter. All analyses were conducted using STATISTICA version 12 (StatSoft, Inc., Tulsa, OK) and Excel 2007 software. To analyse the variables, basic statistical measures were calculated (Table 1). To determine the significance of differences in the average values, one-way analysis of variance and analysis of variance for repeated measures were performed. To determine the significance of differences amongst the age groups in the CSA of the ST and diameter of the ACL, tests for multiple comparisons (Tukey post hoc tests) were performed. *p* values less than or equal to 0.05 were considered statistically significant.

**Table 1:** Basic descriptive statistic

	Age of children’s knees (years)	8	9	10	11	12	13	14	15	16	17	18
**Mean**	Cross-sectional area of ST tendon/femur	0.047	0.0049	0.0062	0.078	0.111	0.082	0.086	0.075	0.088	0.088	0.093
**S.D.**		0.012	0.018	0.012	0.036	0.026	0.042	0.021	0.014	0.020	0.025	0.029
**Mean**	Cross-sectional area of ST tendon/tibia	0.046	0.038	0.054	0.067	0.077	0.102	0.082	0.077	0.079	0.084	0.086
**S.D.**		0.012	0.009	0.022	0.038	0.036	0.033	0.028	0.014	0.019	0.027	0.030
**Mean**	ACL gauge	0.341	0.375	0.464	0.453	0.407	0.493	0.447	0.465	0.518	0.482	0.548
**S.D.**		0.0065	0.086	0.079	0.136	0.078	0.128	0.095	0.104	0.089	0.106	0.110

## Results

The univariate analysis of variance (Table 2) identified significant differences amongst the various age groups in the mean ST CSA at the femoral growth plate level (*p* = 0.00005), the mean ST CSA at the tibial plateau level (*p* = 0.0003) and the ACL diameter (*p* = 0.0023).

**Table 2:** Univariate analysis of variance

Variable	Analysis of variance ANOVA, ***p*** < 0.05000	
	***F***	***p***
**Cross-sectional area of ST tendon/femur**	4.157440	0.000055
**Cross-sectional area of ST tendon/tibia**	3.567452	0.000340
**ACL gauge**	2.942935	0.002363

**Fig. 4:**
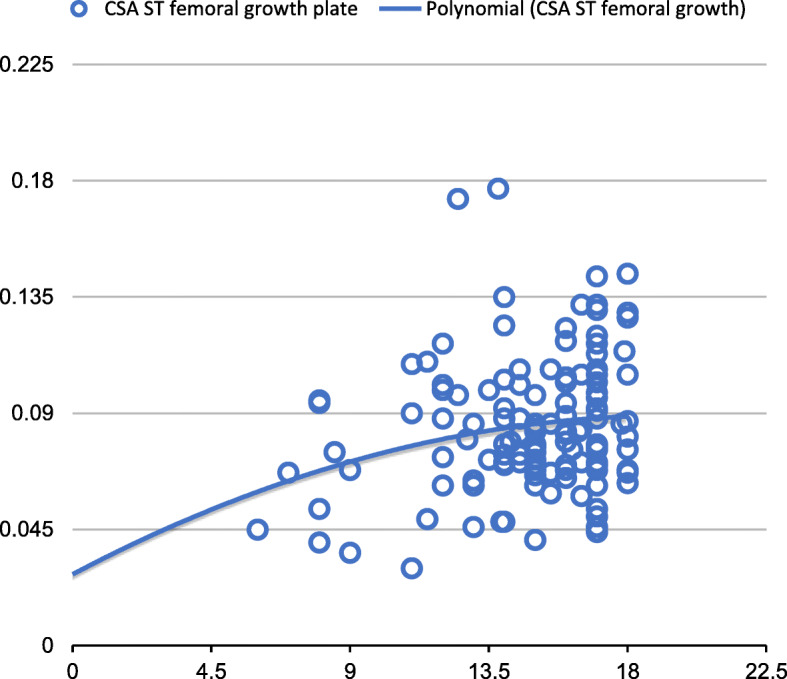
Cross-section area of the ST tendon at the femoral growth plate level—patient age

**Fig. 5:**
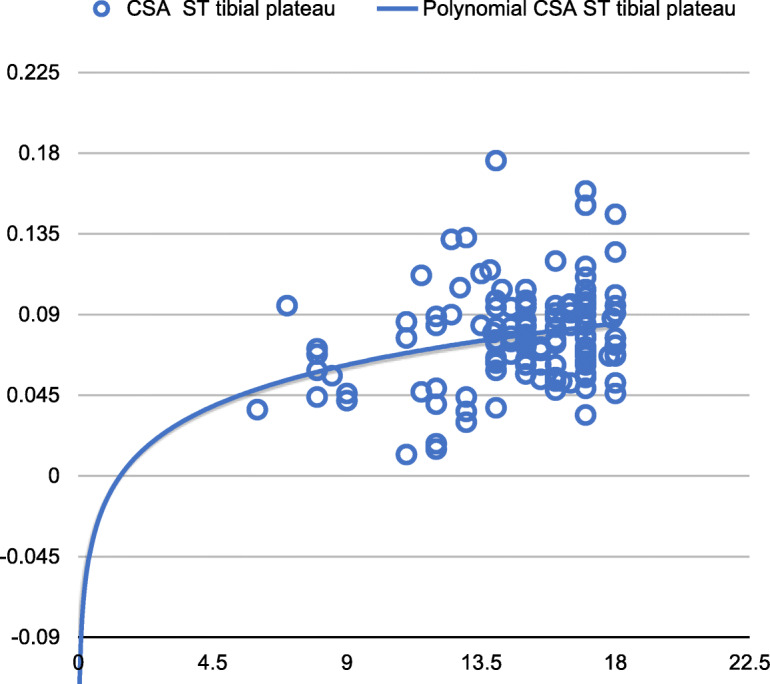
Cross-section area of the ST tendon at the tibial plateau level—patient age

The CSA of the ST at the femoral plate growth level and at the tibial plateau level was related to age (from 8 to 18 years of age), and the growth of the ST CSA was not linear. Analysis of the results led to the conclusion that ST CSA growth progresses symmetrically up to age 12–13 and that the highest growth rate of the CSA of the ST occurred at age 12 and 13 at the level of the femoral growth plate and tibial plateau, respectively. After this age, the increments in the ST CSA were much smaller, and the measurements were comparable to those in adults (Figs. 4 and 5). There was also a correlation between the diameter of the ACL and age. To verify the agreement between assessors, we calculated the intraclass correlation coefficient (ICC) [J. Fleiss et al., Statistical Methods for Rates and Proportions, Willey (2003)] exceeding 0.96 with a confidence level of 0.95 which confirms the consistency of the measurements. We did not find any statistically significant influence of potentially confounding effects such as physical parameters like height or weight of patients.

## Discussion

The most important finding of the present study is that ACL growth proceeds linearly from 8 to 18 years of age. In contrast, we determined that the ST CSA measured at the level of the femoral growth plate and tibial plateau also depends on the patient’s age; the growth was almost complete at the age of 13, even though the growth was not linear. The hypothesis is supported in terms of the characterized relationship between ST CSA growth and child development stage, but the growth of the ACL is not related to the child development stage, especially during puberty.

The number of ACL reconstruction procedures in paediatric patients is increasing. The key indications for ACL reconstruction in paediatric patients are associated with reparable meniscal or osteochondral injuries and recurrent symptomatic knee issues after high-quality rehabilitation or physical activity restriction, which is difficult for children to accept [1, 9, 16]. The aim of surgical ACL reconstruction is to achieve the correct selection, placement and fixation of a graft [2, 17, 18]. The single-bundle, quadrupled hamstring graft is the most common. However, the patellar tendon, quadriceps tendon or Achilles tendon could also be used [8, 17, 19]. Some researchers have suggested using allografts, including those from living parents, for ACL reconstruction in children due to the smaller diameter of hamstrings in children [20]. However, the use of allografts in paediatric ACL reconstruction has had poor clinical outcomes [21]. A hamstring graft offers more advantages than other grafts, including a lower donor site morbidity rate, less anterior knee pain and a shorter electromechanical delay in knee flexion [9].

According to the literature, most researchers planning surgery using an ST-gracilis tendon graft evaluate the hamstring tendon size based on anthropometric measurements, such as age [22] and BMI [10, 12], as well as sports activity and dominant side. It has been documented that only sex [23], height and lower limb length [11, 24] correlate with the hamstring tendon CSA. However, imaging examinations, such as MRI [13–15, 25, 26] and ultrasonography [27], seem to provide the best estimation of tendon size before surgery. While the hamstring CSA can be measured by ultrasound, CT or MRI, of these three methods, MRI shows statistically better results [13]. MRI is clinically useful for predicting the size of the hamstring autograft expected at the time of reconstruction. Additionally, it has been repeatedly confirmed that the intraoperative tendon measurements correlate with preoperative MRI tendon measurements regarding the final ST graft size [12, 13, 28]. Some researchers have drawn attention to the size of the ST before surgery, suggesting that the tendon in question should be at least 7-mm thick or have a minimal CSA ranging from 13.2 to 18 mm^2^ on MRI [29]. According to other researchers, an ST graft with a diameter of less than 7 mm occurred only in 15% of paediatric operations based on a small series of paediatric patients [22]. This outcome is confirmed by our observations concerning the end of the practical growth of the ST CSA at the end of puberty. Researchers have also drawn attention to non-significant differences in ST CSA measurements at the tibial and femoral levels. This finding is confirmed by our study; the present results show no differences in the measurements in question, which is in agreement with the findings of other researchers [28]. Measurement of the CSA of the ST in correlation to the ACL allows for the preoperative preparation of a single, double or quadruple ST surgical procedure. Additionally, MRI allows surgeons to not only plan a well-positioned autograft procedure with adequate fixation to facilitate high-quality rehabilitation but also assess the cartilage growth to avoid complications from growth disturbances [9]. Using MRI enhances surgical planning and helps surgeons present the mode of treatment to the patient and their family [8]. Immature patients have a high risk of reinjury after ACL reconstruction, especially if they return to pivoting sports. One in four patients under 25 years of age sustains a new ACL injury during sports activities. Researchers have emphasized that numerous ACL plasty revisions take place in patients under 20 years of age, at a rate of approximately 14–25%, which is much higher than the corresponding rate in 20- to 25-year-old patients of approximately 0.6–6% [19, 30]. Therefore, presurgical planning and appropriate timing for ACL reconstruction based on the use of the ST as an isolated graft are very important.

There are several limitations to our study. The patients were divided into different age groups (with a 1-year gap between each group), but there was no division based on sex in the knee MRI studies, and we could not obtain reliable measurements of ST length. After modification of the MRI study methodology, the main goal is to obtain data on ST length changes in relation to age.

## Conclusion

ST growth (measured in CSA increments) is almost complete at the age of 13 years, even though the growth is not linear, but ACL growth proceeds linearly from 8 to 18 years of age. MRI is a clinically useful tool for assessing hamstring tendon grafts preoperatively. According to the MRI ST measurements, ACL reconstruction using autologous ST grafts can be performed in children older than 13 years of age.

## Data Availability

Department of Paediatric Traumatology and Orthopedy, Upper Silesian Child Health Centre in Katowice (Poland)

## Abbreviations

ACL: Anterior cruciate ligament
MRI: Magnetic resonance imaging
CT: Computed tomography
CSA: Cross-sectional area
ST: Semitendinosus tendon
